# *TMSB4Y* is a candidate tumor suppressor on the Y chromosome and is deleted in male breast cancer

**DOI:** 10.18632/oncotarget.6743

**Published:** 2015-12-23

**Authors:** Hong Yuen Wong, Grace M. Wang, Sarah Croessmann, Daniel J. Zabransky, David Chu, Joseph P. Garay, Justin Cidado, Rory L. Cochran, Julia A. Beaver, Anita Aggarwal, Min-Ling Liu, Pedram Argani, Alan Meeker, Paula J. Hurley, Josh Lauring, Ben Ho Park

**Affiliations:** ^1^ The Sidney Kimmel Comprehensive Cancer Center, The Johns Hopkins University School of Medicine, Baltimore, MD, USA; ^2^ Veterans Affairs Medical Center, Washington, DC, USA; ^3^ The Georgetown University, Washington, DC, USA; ^4^ George Washington University School of Medicine, Washington, DC, USA; ^5^ The Whiting School of Engineering, Department of Chemical and Biomolecular Engineering, The Johns Hopkins University, Baltimore, MD, USA; ^6^ Present address: Oncology iMED, AstraZeneca, Waltham, MA, USA

**Keywords:** male breast cancer, Y chromosome, TMSB4Y, tumor suppressor, cancer genetics

## Abstract

Male breast cancer comprises less than 1% of breast cancer diagnoses. Although estrogen exposure has been causally linked to the development of female breast cancers, the etiology of male breast cancer is unclear. Here, we show via fluorescence *in situ* hybridization (FISH) and droplet digital PCR (ddPCR) that the Y chromosome was clonally lost at a frequency of ~16% (5/31) in two independent cohorts of male breast cancer patients. We also show somatic loss of the Y chromosome gene *TMSB4Y* in a male breast tumor, confirming prior reports of loss at this locus in male breast cancers. To further understand the function of *TMSB4Y*, we created inducible cell lines of *TMSB4Y* in the female human breast epithelial cell line MCF-10A. Expression of *TMSB4Y* resulted in aberrant cellular morphology and reduced cell proliferation, with a corresponding reduction in the fraction of metaphase cells. We further show that *TMSB4Y* interacts directly with β-actin, the main component of the actin cytoskeleton and a cell cycle modulator. Taken together, our results suggest that clonal loss of the Y chromosome may contribute to male breast carcinogenesis, and that the *TMSB4Y* gene has tumor suppressor properties.

## INTRODUCTION

Male breast cancer is a rare disease that is 100-fold less common than female breast cancer and accounts for less than 1% of all cancers in men [1, 2]. In the United States, approximately 210,000 women and 2000 men will be diagnosed annually with the disease [3, 4]. Globally, male breast cancer has an annual average incidence of <1 case per 100,000 men [5]. Recent epidemiological studies show a slow but steady increase in the annual occurrence of this rare cancer [2, 6, 7].

Due to the low incidence of male breast cancer, most clinical and laboratory research has focused on female breast cancers [8]. However, studies from female breast cancers may not be entirely applicable to men since the etiology of male breast cancer remains unclear. Clinically, breast cancers are traditionally classified according to their receptor status, namely estrogen receptor (ER), progesterone receptor (PR), and HER2 (Human Epidermal Growth Factor Receptor 2). Similar to female breast cancer, the majority of male breast cancers are ER positive and/or PR positive [9]. A notable difference in male breast cancers compared to their female counterparts is the relatively lower percentage of ER, PR, HER2 negative (triple negative) and HER2 positive breast cancers [10–12]. Recent studies also highlight global genomic, transcriptomic, and proteomic differences between female and male breast cancers, with global gene expression profiling showing differences in at least 1,000 genes between female and male breast cancers [13]. In addition, using a computer framework dubbed COpy Number and EXpression In Cancer (CONEXIC), cancer driver genes were shown to be distinct between male and female breast cancers [14].

Although it is unquestionable that exposure to estrogens is a major risk factor for breast cancer development in women, the fact that men can and do develop breast cancer, albeit at much lower incidence, speaks to the fact that other factors likely contribute to breast carcinogenesis. In this study, we hypothesized that the human Y chromosome may be involved in the etiology of male breast cancers. The Y chromosome is one of the smallest human chromosomes, and consists of a minute pseudoautosomal region (PAR) that is homologous to the X chromosome, and a larger male-specific Y region (MSY) [15]. Approximately 450 transcribed genes have been annotated on the Y chromosome, of which 90 are protein-coding [16]. Early studies of male breast cancer genetics have provided some evidence that the Y chromosome may harbor a male specific tumor suppressor since partial or whole Y loss has been reported [17–19]. However, these reports involved small numbers of patients with low resolution genetic techniques. In addition, none of these studies examined Y chromosome *in situ* loss within tumor tissue, hindering the evaluation of clonal Y loss. To our knowledge, there are no reports evaluating *in situ* Y chromosome status in male breast cancers.

In this study, we addressed whether loss of the Y chromosome contributes to male breast carcinogenesis. Using fluorescent *in situ* hybridization (FISH) and droplet digital PCR (ddPCR), our results show clonal Y chromosome loss at a frequency of ~16% (5/31) in two independent cohorts of male breast cancer patients. Furthermore, we observed that Y chromosome loss can occur in ductal carcinoma *in situ* (DCIS) lesions. In order to identify a possible tumor suppressor within the Y chromosome, we used sequence-tagged-site PCR (STS-PCR) in male breast cancer specimens without Y chromosome loss, and show somatic deletion of the *TMSB4Y* gene in a male breast cancer patient, confirming prior reports showing loss of this region. We then created tetracycline-inducible clones of *TMSB4Y* in the human non-tumorigenic female breast epithelial cell line MCF-10A. Our results show that induced expression of *TMSB4Y* led to aberrant morphological changes, persistent reduction in cell proliferation, and a corresponding reduction in the fraction of metaphase cells. Using proximity ligation assays (PLA) and immunoprecipitation with western blotting, we show that *TMSB4Y* interacts directly with β-actin, a main component of the actin cytoskeleton and a modulator of cell cycle progression. Taken together, our results show that *in situ* clonal loss of the human Y chromosome may play an important role in male breast cancer tumorigenesis, and suggest that *TMSB4Y* has tumor suppressive properties.

## RESULTS

### Clonal loss of Y chromosome in male breast cancer is a frequent event

**Figure 1:**
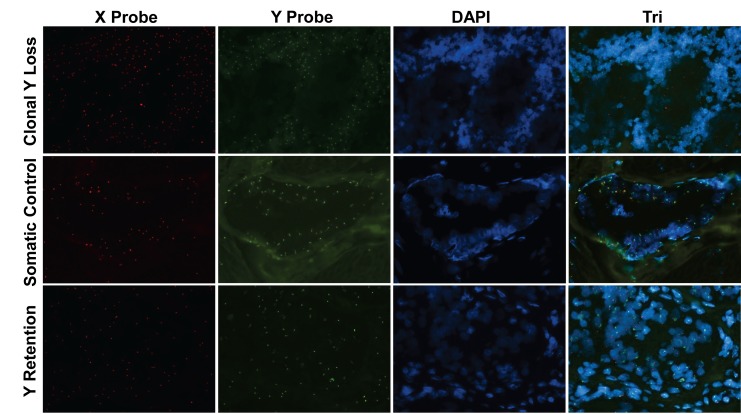
Clonal loss of the Y chromosome in male breast cancer FISH was performed to evaluate Y and X chromosomes on a male breast cancer tissue sample from a patient with clonal Y loss (top panels), normal breast tissue from the same patient as a somatic control (middle panels) and a male breast cancer sample without Y loss (bottom panels). Red, X chromosome FISH Probe; Green, Y chromosome FISH Probe; Blue, DAPI nuclear labeling. Original magnification: 20X.

To address the hypothesis that Y chromosome loss may have a role in breast carcinogenesis, we first evaluated its loss in male breast cancers. We obtained FFPE tissue blocks of male breast cancers from 15 patients (cohort 1, Table 1) and used these to synthesize a tissue microarray (TMA). This TMA was then analyzed for Y chromosomal loss by FISH, along with an X chromosome FISH probe as a control (Figure 1). In order to survey the entire Y chromosome, we used various combinations of FISH probes specific for the short arm, centromere, and long arm of the Y chromosome (Figure S1). By these criteria, we observed clonal loss of the whole Y chromosome in 2 out of 15 (~13.33%) male breast cancer patients.

**Table 1:** Clinical characteristics of patients The five Y loss cases are highlighted. nd, not done; na, not available.

Cohort 1 Patient	ER	PR	HER2	Stage
01	+	+	−	IA
02	+	+	nd	IA
03	+	+	nd	IA
04	+	+	nd	IA
05	+	+	−	IA
06	+	+	nd	IA
07	+	Focally +	Weakly +	IIIC
08	+	+	nd	IIA
09	+	+	nd	IIA
10	+	+	Equivocal	IIB
11	+	−	−	IIIA
12	nd	nd	nd	IA
13	+	−	−	IIA
14	+	−	−	IB
15	na	na	na	na
**Cohort 2** **Patient**				
01	+	+	na	IIIB
02	+	nd	na	1
03	+	+	na	na
04	+	+	na	IIIB
05	nd	nd	na	DCIS
06	nd	nd	na	DCIS
07	+	+	+	IIIB
08	+	+	na	IIA
09	+	+	na	IIA
10	+	+	na	IA
11	+	+	na	IIB
12	na	na	na	IV
13	+	+	na	IIA
14	+	+	na	IA
15	+	+	na	IIA
16	+	−	na	IA
17	+	−	nd	IV

Next, we obtained 19 additional male breast cancer FFPE samples, however, only 17 had adequate tissue for analysis (cohort 2, Table 1). Because blocks were unavailable for these samples, we could not create a separate TMA. Therefore, we analyzed each specimen by FISH (Table 2), and demonstrated clonal somatic Y loss in 3 additional patients. Due to DNA degradation and limited material, 3 of 17 samples were inadequate for FISH yielding an overall Y loss frequency of 17% (5 of 29 patients combining both cohorts). In order to analyze the remaining 3 samples that were unevaluable by FISH, we performed ddPCR on FFPE DNA using Taqman probes and primers specific to the X and Y chromosomes to assay for Y chromosome loss. As we have previously reported, fragmented DNA is optimal for ddPCR and minute amounts of DNA can be used for accurate quantification of alleles using FFPE derived material [20]. As seen in Table 2, the ratio of Y versus X positive signals (ratio Y/X) as measured by ddPCR was first validated using a commercial source of female and male control gDNA, with ratios of 0 and 0.966, respectively. We also included as a control a patient from cohort 1 with Y loss that showed a Y/X ratio of 0.193, supporting that ddPCR could be used to assess Y loss. As seen in Table 2, due to variability in tumor cellularity and non-tumor normal tissue contamination, tumors with Y loss generally had a Y/X ratio of less than 0.200, though the exception was patient 5 who had a Y/X ratio of 0.410, likely due to a higher than observed amount of normal tissue contamination. Notably, we observed two cases, patients 3 and 4, with Y/X ratios of 0.354 and 0.383 suggestive of Y loss. However, for these two patients, FISH analysis showed Y retention with somatic duplication of the X chromosome, yielding an artificially lower Y/X ratio by ddPCR. These results highlight some of the potential pitfalls and caveats of allelic enumeration and ratios, and that *in situ* analysis is often needed for definitive conclusions. Although one of the three patients with unevaluable samples for FISH yielded an equivocal result (patient 6), two patients clearly had Y/X ratios demonstrating retention of Y, though X loss could not be excluded in these patients (patients 14 and 16). Thus, combining results from the two independent cohorts of male breast cancer patients using both FISH and ddPCR, we observed a Y loss frequency of ~17% (5 of 29 male breast cancer patients), though if patients 14 and 16 are included via the ddPCR results, the frequency is marginally decreased to ~16% (5 of 31 patients).

**Table 2:** Y chromosome loss in male breast cancer patients

Patient Number	Tumor Cellularity (%)	Positive ddPCR Droplets		Ratio Y/X	ddPCR Interpretation	FISH
		Y Chr Taqman	X Chr Taqman			
01	70	7	524	0.013	Y Loss	Y Loss
02	60	6	114	0.053	Y Loss	Y Loss
03	80	357	1009	0.354	Equivocal	Y Retention *
04	80	217	566	0.383	Equivocal	Y Retention *
05	60	84	205	0.410	Equivocal	Y Loss
06	60	54	116	0.466	Equivocal	Unevaluable
07	70	47	73	0.644	Y Retention	Y Retention
08	70	17	24	0.708	Y Retention	Y Retention
09	70	158	196	0.806	Y Retention	Y Retention
10	80	57	52	1.096	Y Retention	Y Retention
11	70	134	117	1.145	Y Retention	Y Retention
12	70	334	214	1.561	Y Retention	Y Retention
13	70	482	293	1.645	Y Retention	Y Retention
14	60	156	91	1.714	Y Retention	Unevaluable
15	80	2067	1188	1.740	Y Retention	Y Retention
16	50	53	23	2.304	Y Retention	Unevaluable
17	70	252	86	2.930	Y Retention	Y Retention
Cohort 1 patient with Y loss	80	101	523	0.193	Y Loss	Y Loss
Male gDNA	NA	2628	2720	0.966	Y Retention	Male Control
Female gDNA	NA	0	3451	0.000	No Y	Female Control

Tumor gDNA was extracted from FFPE tissue slides from male breast cancer patients and analyzed using ddPCR Taqman probes specific for X and Y chromosomes. Fourteen of 17 ddPCR-analyzable samples were validated by FISH; three patients had FFPE tissue that could not be validated with FISH.

* Denotes male breast cancer patients who have duplication of X chromosome.

For one of the patient samples with Y loss in cohort 1, a corresponding DCIS sample was available (Figure S2A), presenting an opportunity to ascertain whether Y loss occurred prior to invasive disease. As shown in Figure S2B, the DCIS from this patient was confirmed as non-invasive using an anti-smooth muscle actin stain. We further showed via FISH that this lesion had somatic Y loss, with the adjacent stromal tissue retaining the Y chromosome as an internal control (Figure S2C).

### The Y chromosome gene *TMSB4Y* is lost in male breast cancer

Although total Y loss was present in ~16% of our patient samples, we reasoned that if there was a tumor suppressor on the Y chromosome, deletion of this candidate tumor suppressor could occur with retention of the remainder of the Y chromosome. Microdeletions within the Y chromosome have been reported in males with varying degrees of frequency [21–23]. To further investigate this, we extracted gDNA from five male breast cancer patients who retained the Y chromosome, and amplified 33 Sequence Tagged Sites (STSs) within the MSY using standard PCR (Table S1, Figure S3). Primers were designed using published data for these genomic loci [24]. Paired tumor vs normal gDNA (identified by the study pathologist) were extracted from FFPE slides, but 2 of 5 samples had inadequate gDNA for further analysis. The STS primer pair S27 was not successfully amplified in any tumor or normal samples, likely due to fact that this amplicon size is ~1kb, and could not be amplified from fragmented FFPE gDNA.

**Figure 2:**
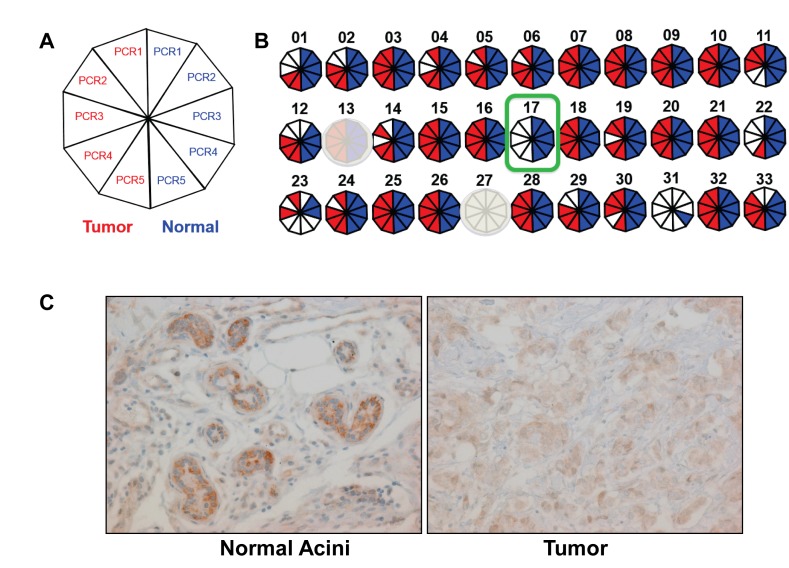
*TMSB4Y* is lost in male breast cancer and expressed in normal male breast tissue **A**. Regional Y loss was assessed by STS-PCR that was repeated 5 times using 5 serial slides from matched tumor and normal FFPE gDNA from three patients as described in the text. A summary of each STS-PCR is depicted as a decagon, where a positive reaction is red for tumor, and the corresponding normal somatic control is blue. **B**. Results summary for a patient who retained the Y chromosome in his breast cancer by FISH, but demonstrated consistent loss of the STS-PCR marker S17, a region containing the *TMSB4Y* gene, in tumor but not in adjacent normal tissue (outlined in green). Primer sets S13 and S27 were uninformative (shaded out) due to non-specific amplicons and lack of amplicons (amplicon size too large as described in text), respectively. **C**. Immunohistochemistry labeling for *TMSB4Y* was performed on normal breast tissue (left) and corresponding breast cancer (right) from a male breast cancer patient with known Y chromosome loss by FISH. Original magnification: 20X.

From the remaining 32 STS primer pairs, S17 did not amplify for the tumor gDNA for one of the three analyzable male breast cancer patients, but was present in the germline paired normal gDNA (Figure 2B). This primer pair amplifies a region including exon 1 and intron 1 of *TMSB4Y*, a gene encoding the actin sequestering protein, Thymosin Beta 4, Y-linked. Prior literature also demonstrated that this region was lost in male breast patients with a frequency of 40% [25]. Furthermore, a query of the cBioPortal database using the parameter “TMSB4Y: HOMDEL” yielded a data set that shows *TMSB4Y* deletion in 16% (10/59) of metastatic prostate adenocarcinomas (Figure S4) [26]. To investigate whether *TMSB4Y* expression is consistent with a tumor suppressor, we sought to determine *TMSB4Y* expression in normal male breast tissue. We performed immunohistochemistry (IHC) to assess TMSB4Y protein expression in normal male breast tissue, using matched breast tumor tissue with Y loss as a negative control. Because *TMSB4Y* has a homolog located on the X chromosome, *TMSB4X*, we initially tested the specificity of the antibody. We transiently transfected *TMSB4X* and *TMXB4Y* cDNAs separately into HEK293 cells and harvested lysates for western blot. Unfortunately, none of our TMSB4X antibodies could detect TMSB4X protein, and therefore we obtained a FLAG-tagged *TMSB4X* cDNA and repeated the experiment. As shown in Figure S5, the TMSB4Y antibody specifically detected the TMSB4Y protein by western blot analysis but did not detect TMSB4X.

We then performed IHC on our tissue sample. As shown in Figure 2C, normal breast epithelial cells labeled with strong intensity compared to the adjacent tumor tissue, which demonstrated no labeling above a low level of nonspecific background. These results show that *TMSB4Y* is expressed in normal male breast tissue and its expression is lost in a patient with known Y chromosome loss. Unfortunately, the TMA and tissue slides of MBC patients (both cohort 1 and 2) were exhausted and we could not perform IHC to evaluate *TMSB4Y* expression in our other MBC patients.

### *TMSB4Y* expression alters breast epithelial cell morphology

**Figure 3:**
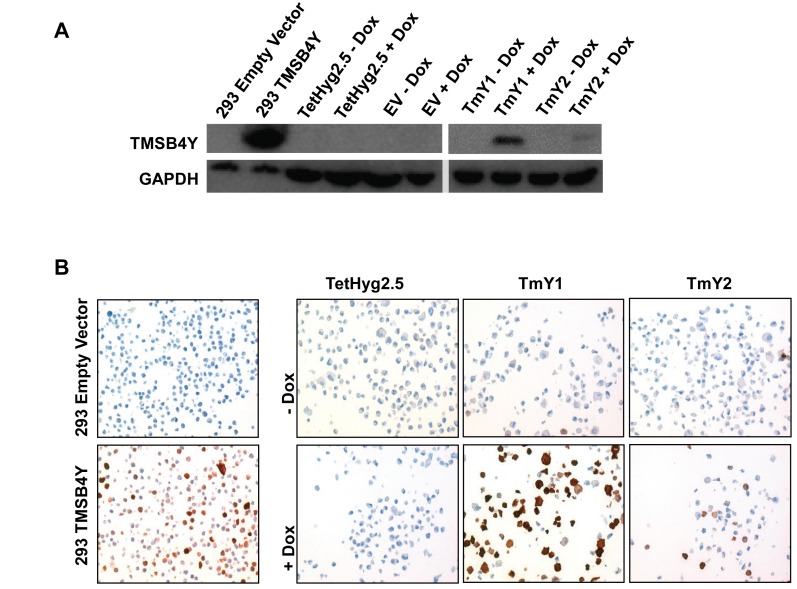
TMSB4Y expression in Dox-inducible clones The *TMSB4Y* cDNA was cloned into the pBI-EGFP vector and stably transfected into the Dox-inducible MCF-10A cell line, TetHyg2.5, to the create *TMSB4Y* Dox-inducible clones TmY1 and TmY2. HEK293 cells were transiently transfected with empty vector (293 Empty Vector) or a *TMSB4Y* cDNA (293 TMSB4Y) to serve as negative and positive antibody controls, respectively. Parental TetHyg2.5, a stably transfected empty vector (EV) control cell line and the two inducible clones, TmY1 and TmY2, were placed in un-induced (−Dox) and induced (+Dox) media conditions for 48 hours prior to harvesting for lysates and fixation for FFPE blocks. TMSB4Y protein expression was verified via **A**.) western blotting using an anti-TMSB4Y antibody and GAPDH antibody as loading control, and **B**.) immunohistochemistry labeling of FFPE cell pellets using an anti-TMSB4Y antibody and counterstaining. Original magnification: 20X.

To investigate the effects of *TMSB4Y* in breast cells, we generated stable Dox-inducible clones in the MCF-10A cell line [27]. MCF-10A is a genetically stable non-tumorigenic breast epithelial cell line derived from reduction mammoplasty tissue, and a derivative cell line, TetHyg2.5, was created by stable transfection of the Tet repressor protein. MCF-10A cells are ideal for these studies, since they are non-cancerous, and therefore have less concern regarding pre-existing genetic alterations that could mask any given phenotype. In addition, they are derived from a female, so concerns regarding TMSB4Y expression are not applicable. *TMSB4Y* was cloned into the bidirectional pBI-EGFP vector, which contains the Tet response element, and expresses green fluorescent protein (GFP) simultaneously with *TMSB4Y*. We generated TmY1 and TmY2, two Dox-inducible cell lines that express both GFP and *TMSB4Y* when induced with Dox. A control cell line was also created called EV, which contains the pBI-EGFP empty vector and expresses only GFP when induced with Dox. Expression of *TMSB4Y* after Dox-induction was verified via western blotting (Figure 3A) and immunohistochemistry (IHC) on tissue cellblocks made from these cell lines (Figure 3B). We noted that TmY1 showed stronger induction of *TMSB4Y* compared to TmY2.

**Figure 4:**
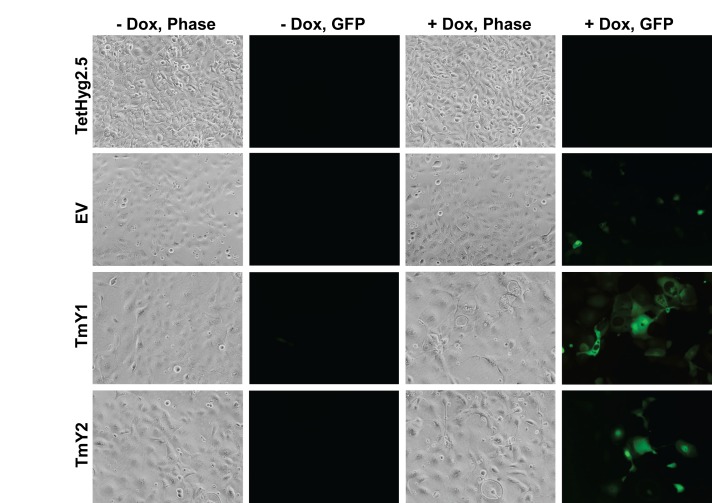
TMSB4Y expression leads to changes in cell morphology Parental TetHyg2.5, a stably transfected empty vector (EV) control cell line and the two TMSB4Y Dox-inducible clones, TmY1 and TmY2, were placed in un-induced (−Dox) and induced (+Dox) media conditions for 48 hours and then imaged using phase contrast (Phase) and fluorescence (GFP) microscopy. Note the enlarged and aberrant cellular borders present in Dox-induced *TMSB4Y* clones, TmY1 and TmY2. Original magnification: 20X.

After Dox-induction of *TMSB4Y,* we observed morphologic changes in TmY1 and TmY2 cells (Figure 4), with cells displaying a flattened appearance with abnormal borders. Although EV control cells did not show morphology changes, we used as an additional control transient overexpression of GFP in MCF-10A cells and found this was non-toxic (Figure S6). Additionally, we performed immunofluorescence with antibodies against F-actin to better visualize the cellular architecture. As seen in Figure S7, F-actin labeling after Dox-induction confirmed aberrant cell morphology seen in TMSB4Y expressing cells. Because male breast cancers are most often ER positive, and MCF-10A are ER negative cells, we wanted to evaluate expression of TMSB4Y in an ER positive breast cancer cell line. As such, we then transiently co-expressed the *TMSB4Y* cDNA with GFP in the human breast epithelial adenocarcinoma cell line MCF-7. As seen in Figure S8, similar aberrant morphological changes were observed after 48hrs in GFP positive cells.

### TMSB4Y expression reduces cell proliferation

**Figure 5:**
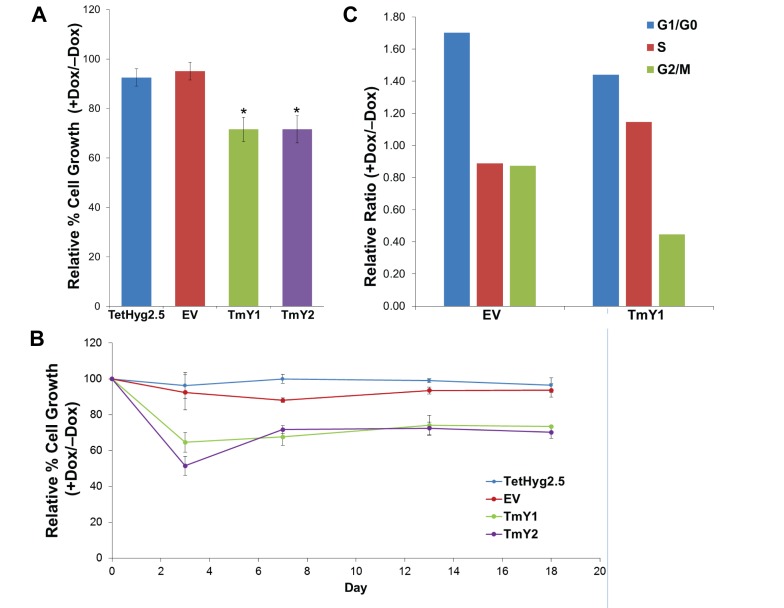
TMSB4Y expression reduces cell proliferation Parental TetHyg2.5, a stably transfected empty vector (EV) control cell line and the two TMSB4Y Dox-inducible clones, TmY1 and TmY2, were placed in un-induced (−Dox) and induced (+Dox) media conditions and used for cell proliferation assays and FACS analysis. **A**. TmY1 and TmY2 exhibited ~30% of reduced proliferation upon Dox induction (*p < 0.05) relative to un-induced cells after 6 days of growth. **B**. Relative reduced cell proliferation after Dox-induction in both TmY1 and TmY2 was observed with prolonged culture as measured by cell counts at Days 3, 7, 13, and 18. **C**. *TMSB4Y* expression reduced the relative metaphase cell fraction in TMSB4Y expressing cells (TmY1) after Dox induction, but not in empty vector (EV) control cells after 48 hours and GFP-sorting for cell cycle analysis.

Tumor suppressors often reduce the proliferation of cells when expressed. In order to assess this phenotype, we compared proliferation rates of our cell lines with and without TMSB4Y expression. Dox-induction of *TMSB4Y* significantly reduced the growth rates of TmY1 and TmY2 by approximately 30% when compared to TetHyg2.5 and EV (P < 0.05) in a 6-day growth assay (Figure 5A). This reduction in proliferation was persistent, as observed in a detailed growth assay where cell counts were taken on days 0, 3, 7, 13, and 18 (Figure 5B).

To further understand the mechanism of reduced proliferation, we then performed fluorescence activated cell sorting (FACS) analysis on our cells. FACS was used to sort out the GFP-positive TmY1 cells 48hrs after Dox-induction, and then these GFP-positive cells underwent cell cycle analysis based on their DNA content. TmY2 cells could not be used for these experiments due to the low level of GFP inducible expression. *TMSB4Y* expression in these GFP-positive cells sharply reduced the metaphase fraction (G2/M) in TmY1 when compared to EV (Figure 5C). This metaphase reduction correlates with the persistent reduction in cell proliferation observed, suggesting that *TMSB4Y* expression modulates cell cycle progression.

### *TMSB4Y* interacts with β-actin

**Figure 6:**
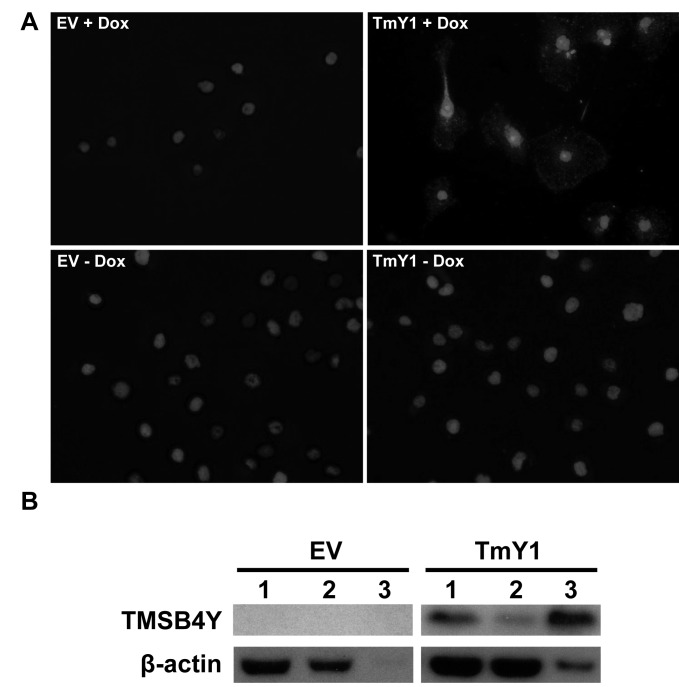
TMSB4Y interacts with β-actin The empty vector control (EV) and TmY1 were grown in un-induced (−Dox) and induced (+Dox) conditions for 48 hours prior to assaying for TMSB4Y and β-actin protein interactions via **A**. a proximity ligation assay (PLA) using antibodies against *TMSB4Y* and β-actin as described in the text. The red signals (top right panel) demonstrate a *TMSB4Y*-β-actin interaction based on their *in situ* proximity of 0 to 40nm. Nuclei of cells are stained with DAPI (blue). Original magnification: 20X. **B**. Cell lysates were also used for immunoprecipitation (IP) experiments using a *TMSB4Y* antibody on Dox-induced lysates from EV and TmY1. The TMSB4Y antibody was used for IP, and then total lysate (1), supernatant after IP (2) or the IP fraction (3) was stained via western blot using anti-TMSB4Y and anti-β-actin antibodies.

Thymosin beta proteins have been identified as actin monomer sequestering proteins in mammalian cells [28, 29]. However, to our knowledge, there are no studies demonstrating that *TMSB4Y* directly interacts with actin. We reasoned that *TMSB4Y* might modulate cell cycle progression through a possible interaction with actin, a main component of the cytoskeleton [30]. In support of this, the intracellular actin cytoskeletal state has been shown to regulate cell cycle progression via retrograde signaling [31, 32]. To demonstrate a TMSB4Y interaction with actin, we performed a proximity ligation assay (PLA) using antibodies specific to TMSB4Y and β-actin. PLA employs two specific antibodies of interest, each containing a linked nucleic acid sequence such that proteins within 0 to 40nm of one another can then be ligated via their nucleic acid sequences, with subsequent amplification and fluorescence detection. Using PLA, we observed that TMSB4Y and β-actin are in close proximity *in situ* only after Dox-induction, supporting a direct protein-protein interaction (Figure 6A). We further confirmed this direct interaction between TMSB4Y and β-actin by immunoprecipitating β-actin with an anti-TMSB4Y antibody in Dox-induced TmY1 lysates, however, this interaction was absent in Dox-induced EV lysates (Figure 6B). Together, these results suggest that TMSB4Y directly interacts with β-actin, and that this interaction may lead to the aberrant morphology and decreased proliferation seen upon TMSB4Y expression.

## DISCUSSION

Cancer is a disease caused by a successive series of genetic alterations that lead to selective proliferative advantages in a single cell, which then expands into a tumorigenic clone [33]. Theoretically, all cells that originate from this clone will retain specific genetic “hits” acquired in this tumorigenic process. Therefore, our report of clonal *in situ* loss of the Y chromosome in ~16% of male breast cancers adds to the weight of evidence that Y loss contributes to male breast carcinogenesis. In accord with the clonal progression theory, we also observed clonal Y loss in a corresponding DCIS lesion from a male breast cancer patient, suggesting that Y loss can be an early event in male breast cancer, further supporting a role for early tumor formation.

We demonstrated that clonal Y loss was present in a percentage of male breast cancers via FISH. However, the capacity to perform FISH on FFPE tissues is variable due to the quality of DNA from this analyte. Therefore, we used ddPCR to determine Y loss in male breast cancer samples that were inadequate for FISH analysis. In theory, the loss of Y would skew the Y/X ratio towards zero. Previously, in an analysis of loss of heterozygosity of *BRCA2* alleles, we showed that a basal level of contamination from surrounding normal stromal cells could be overcome using the highly sensitive and specific platform of ddPCR [20]. However, although normal cellular contamination is a common issue, X chromosomal duplication can also occur, as in 2 of our patients, making ddPCR less reliable to assess Y loss. However, our results do suggest that Y retention can be reliably concluded based upon the ratio of Y/X positive droplets using ddPCR. Thus, we included two of these patients in our overall assessment.

Our results showing Y loss support recent studies suggesting that the human Y chromosome has tumor suppressive functions. For example, mosaic loss of Y chromosome in peripheral blood is associated with a higher risk of non-hematologic cancers in men [34]. Furthermore, this loss of Y in peripheral blood is associated with smoking in a dose-dependent manner [35], suggesting a possible link with smoking and Y loss. Additionally, male microchimerism in women has been shown to reduce the risk of breast cancer by approximately one third [36]. In fact, male chimerism observed in the peripheral blood of women generally increases the survival of women affected by cancers and cardiovascular diseases [37]. Male microchimerism is a phenomenon that occurs in pregnant women with male fetuses, with subsequent exchange of fetal and maternal cells via the placenta and persistence of fetal cells [38]. In addition, a previous study demonstrated that transfer of the Y chromosome into a prostate cancer cell line with Y loss suppressed formation of xenografts in 59 out of 60 athymic nude mice [39]. Collectively, these previously described reports combined with our data strongly suggest that the human Y chromosome harbors tumor suppressive properties.

Upon observing Y loss in 5 out of 31 (~16%) of our male breast cancer patients, we hypothesized that male breast cancer without Y loss may still have deletion of a candidate tumor suppressor gene. Therefore, we utilized STS-PCR to investigate regional Y loss in male breast cancer patients with Y chromosome retention. Using STS-PCR, we showed somatic loss of an STS containing the *TMSB4Y* gene in one of three of our male breast cancer patients with Y retention (Figure 2A). A previous study using array comparative genomic hybridization (aCGH) demonstrated a 40% (10/25 patients) deletion of the Yq11.1-q11.221 locus in male breast cancer, a region that contains *TMSB4Y* [25]. Furthermore, an analysis of public databases via cBioPortal showed that *TMSB4Y* was deleted in 16% (10/59) prostate cancer samples (Figure S4) [40]. These combined data further support that *TMSB4Y* is a tumor suppressor whose loss contributes to male breast cancer.

Functional analysis of *TMSB4Y* also suggests this gene has tumor suppressor properties. When *TMSB4Y* is overexpressed in a Dox-inducible MCF-10A background, we observed altered cell morphology, reduced cell proliferation, and a reduced G2/M population. Mechanistically using PLA and immunoprecipitation, we showed that *TMSB4Y* directly interacts with β-actin. Since β-actin is a main component of the actin cytoskeleton, a major factor in cell morphology and a regulator of cell cycle progression [31, 32], the direct interaction of *TMSB4Y* and β-actin may modulate the actin cytoskeletal turnover in cells, and in turn could alter cell morphology with subsequent retardation of cell cycle progression.

In conclusion, we observed that ~16% of male breast cancers demonstrate *in situ* clonal loss of the Y chromosome. These data and those of others, support our hypothesis that the human Y chromosome may be a tumor suppressor in male breast cancer. Furthermore, the Y chromosome gene *TMSB4Y* may be a tumor suppressor gene that normally functions through its direct interaction with β-actin, which in turn regulates cell morphology and cell proliferation. These results lend new insights into the potential role of the Y chromosome as a tumor suppressor and implicate *TMSB4Y* in the etiology of male breast cancers.

## MATERIALS AND METHODS

### Formalin fixed paraffin embedded (FFPE) tissue of male breast cancer patients

Thirty-two male breast cancer samples were collected from 2 independent cohorts. Cohort 1 consists of 15 male breast cancer patients from Johns Hopkins Hospital collected from 1990 to 2013. FFPE tissue blocks were available and samples were used to create a tissue microarray (TMA) for analysis under an IRB approved protocol. Cohort 2 consists of samples from 19 male breast cancer patients from the Veterans Affairs Medical Center, Washington DC collected between 1996 to 2012. The study was approved by the IRB, VAMC DC. We were able to extract genomic DNA from 17 of these 19 FFPE samples that was suitable for droplet digital PCR (ddPCR), though only 14 had suitable quality and quantity for both ddPCR and fluorescence *in situ* hybridization (FISH).

### FISH

FFPE tissue slides were de-paraffinized and washed with xylene and EtOH. Slides were pretreated, hybridized with FISH probes, and then mounted for microscope observation. Pretreatment was performed using Pretreatment Kit I (02J02-032, Abbott Molecular). Probes were mixed with LSI/WCP Hybridization Buffer (06J67-001, Abbott Molecular). Slides were counterstained with DAPI, and mounted with ProLong® Gold (P36930, Invitrogen). All fluorescence microscopy photos were imaged with NIS-Elements BR2.30. All FISH probes used were from Abbott Molecular: Vysis CEP X (DXZ1) SO Probe (centromeric, 05J08-033), Vysis CEP X (DXZ1) SA Probe (centromeric, 05J09-033), Vysis CEP Y (DYZ1) SGn Probe (q arm, 05J10-034), Vysis CEP Y (DYZ3) SO Probe (centromeric, 05J08-035), and Vysis LSI SRY SO Probe (p arm, 05J27-089).

### Droplet Digital PCR (ddPCR)

As previously described, ddPCR was performed using the QX100™ Droplet Digital PCR System according to the manufacturer's recommendations (Bio-Rad) [41]. TaqMan® Copy Number Assays (Life Technologies) included Hs00314226_cn (FAM labeled) and Hs04125506_cn (VIC labeled) probes. Control genomic DNA used for these studies were commercially purchased male (Promega, G1471) and female (Promega, G1521) DNA. ddPCR was performed with Bio-Rad's recommended two-step thermo-cycling protocol with a 58°C annealing/extension step. All data analysis was performed using QuantaSoft (Bio-Rad).

### Genomic DNA (gDNA) extraction from FFPE tissue slides

Tumor and normal areas were identified and marked by the study pathologist (P.A.). Tissue slides were de-paraffinized and pinpoint solution (D3001-1, Zymo Research) was applied specifically onto the marked areas. DNA was then extracted using the QIAmp DNA FFPE tissue kit (56404, Qiagen) as per the manufacturer's protocol.

### STS-PCR

The male specific region of the Y chromosome Breakpoint mapper [24] was used to analyze the sequence-tagged sites (STS) status of gDNA extracted from patients. Thirty-three sets of STS primers across the MSY region (Table S1) were used. Each set of STS-PCR was repeated 5 times for reproducibility.

### Cell culture

TetHyg2.5, a derivative of the non-tumorigenic human breast epithelial cell line MCF-10A [27] was grown in DMEM/F12 (1:1) supplemented with insulin at 10 μg/mL, hydrocortisone at 0.5 μg/mL, and cholera toxin at 0.1 μg/mL (hereafter denoted as “supplemented DMEM/F12”), 5% horse serum (Gibco), EGF at 20 ng/mL, and hygromycin B at 14.3 μg/mL. Dox-inducible TetHyg2.5 derivatives (EV, TmY1, TmY2, and UA3) were grown in supplemented DMEM/F12 with 5% Tetracycline (Tet)-free FBS (HyClone), EGF at 20 ng/mL, hygromycin B at 7.15 μg/mL, and G418 at 120 μg/mL. HEK293 and MCF-7 cells were grown in DMEM media with 5% FBS. All supplements were purchased from Sigma-Aldrich unless otherwise specified. MCF-10A, HEK293, and MCF-7 cells were purchased from American Type Culture Collection (ATCC).

### Plasmids and transfections

The *TMSB4Y* cDNA was purchased as pCMV6-XL5-TMSB4Y (Origene) and was subcloned into the pBI-EGFP vector (Clontech). The *TMSB4X* cDNA was purchased as tagged and untagged plasmids as pCMV6-Entry-TMSB4X and pCMV6-XL5-TMSB4X, respectively (Origene) and used for transient transfection experiments in HEK293 cells. Plasmids were amplified using the Qiagen Hi-Speed Maxi kits (Qiagen) as per the manufacturer's protocol. Transient transfections were carried with FUGENE6 (Promega) as per the manufacturer's protocol.

### Generation of Dox-inducible clones

TetHyg2.5 cells were seeded on day 0 and transfected with a cDNA of *TMSB4Y* cloned into the pBI-EGFP vector and a neomycin resistance vector on day 1. Selection media (hygromycin B at 7.15 μg/mL, and G418 at 120 μg/mL) was used from day 2 onwards. On day 5, cells were plated into 96-well plates and observed for clones expressing green fluorescent protein (GFP) upon 48-hr doxycycline (Dox)-induction at 2ug/ml. Two clones, TmY1 and TmY2, were identified as Dox-inducible cell lines, and an empty vector control (EV) was also generated.

### Immunoblotting

Whole-cell protein lysates were prepared using Laemmli sample buffer and resolved by SDS-PAGE using NuPAGE gels (Invitrogen), transferred to a 0.2 μm pore size Invitrolon polyvinylidene difluoride (PVDF) membranes (Invitrogen), and probed with primary antibody followed by incubation with horseradish peroxidase-conjugated secondary antibodies. Antibodies used include anti-*TMSB4Y* (clone 6G4) mouse monoclonal antibody (SAB1403013, Sigma Aldrich), anti-FLAG M2 antibody (200472-21; Agilent), anti-rabbit IgG HRP-linked antibody (7074, Cell Signaling Technology), anti-mouse IgG HRP-linked antibody (7076, Cell Signaling Technology), anti-GAPDH (D16H11) XP rabbit monoclonal antibody (5174, Cell Signaling Technology), and anti-β-actin (clone 13E5) rabbit monoclonal antibody (4970, Cell Signaling Technology).

### Cell line tissue block

Cells were trypsinized and fixed in 10% buffered formalin overnight. Cells were then centrifuged and resuspended with 1X PBS, then mixed via pipetting with 2% agarose solution. Agar plugs were processed into FFPE tissue blocks and slides.

### Immunohistochemistry (IHC)

IHC was performed using the PowerVision + Poly-HRP anti-Mouse IHC Detection System (Immunovision). Briefly, slides were steamed in EDTA solution and incubated with anti-smooth muscle actin antibody mouse monoclonal (1:800 dilution, DAKO, m0851) or anti-*TMSB4Y* (clone 6G4) mouse monoclonal antibody (1:200 dilution, SAB1403013; Sigma Aldrich). Poly-HRP anti-mouse IgG antibody was applied and then visualized with 3,3′-diaminobenzidine (Sigma). Slides were counterstained with hematoxylin.

### Cell proliferation assay

Cells were seeded in supplemented DMEM/F12 with 1% Tet-free FBS (HyClone), EGF at a physiological concentration (0.2 ng/mL), hygromycin B at 7.15 μg/mL, and G418 at 120 μg/mL. Dox induction was performed by seeding cells in their respective media with 2μg/mL of Dox. 2 × 10^4^ cells per well of a 6-well tissue culture dish were seeded on day 0. Media was changed every third day, and cells were harvested for cell counting on days 3, 6, 7, 13, and 18 using a Beckman Coulter® Vi-CELL™ XR Cell Viability Analyzer.

### Proximity ligation assay (PLA)

Cells were seeded in chamber slides and Dox for induction x 48 hours. Slides were fixed in cold methanol for 15min in −20°C and treated with cold acetone for 1 minute at room temperature. Mouse monoclonal anti-*TMSB4Y* antibody (Clone 6G4, SAB1403013, Sigma Aldrich) at 1:200 and rabbit monoclonal anti-β-actin antibody (Clone 13E5, 4970, Cell Signaling Technology) at 1:200 were incubated overnight. PLA was performed as per instructions of the DuoLink® In Situ Red Starter Kit Mouse/Rabbit (DUO92101, Sigma Aldrich).

### Immunoprecipitation

Cells were rinsed with ice-cold PBS, scraped, and lysed on ice in lysis buffer containing 1% Triton X-100, 10% glycerol, 100mM NaCl, 50mM Hepes (pH 7.2), 10mM NaF (all from Sigma Aldrich), 10mM Na_3_VO_4_ (New England Biosciences), and minitab cOmplete^TM^ protease inhibitor with EDTA (Roche). Lysates were quantified using the BCA protein assay reagent (Pierce). Immunoprecipitation was performed by incubating 1mg of lysate with 1μg of anti-*TMSB4Y* antibody (Clone 6G4, SAB1403013, Sigma Aldrich) overnight at 4^°^C. Lysates were then incubated with Dynabeads Protein G (Life Technologies) for 4 hours at 4^°^C. Dynabeads were then washed with cold lysis buffer and boiled at 100^°^C for 5 minutes in 2x Laemmli buffer before immunoblotting.

### Cell-cycle analysis with flow cytometry

Cells were seeded at 50% confluency in T75 flasks and Dox-induced for 48hrs. GFP positive cells were isolated by fluorescence activated cell sorting using BD FACSAria II and fixed in PBS/3% formaldehyde/0.4% NP-40 containing 2 mg/mL Hoechst 33258 (Invitrogen). DNA content was measured with a BD LSR flow cytometer (BD Biosciences), and percentages of G1/G0, S, and G2/M phase cells were determined using Modfit LT software (Verity Software House).

### Statistical analysis

All statistical analyses were conducted with GraphPad Prism software. For the t-tests conducted, p<0.05 was considered significant.

## SUPPLEMENTARY MATERIAL FIGURES AND TABLE


