# Supplementary material for: TMSB4Y is a candidate tumor suppressor on the Y chromosome and is deleted in male breast cancer
Source: Oncotarget. 2015 Dec 23;6(42):44927–40. doi: 10.18632/oncotarget.6743 (PMC4792601; doi:10.18632/oncotarget.6743)
Supplement: Supplementary file 1 [file oncotarget-06-44927-s001.pdf]

TMSB4Y is a candidate tumor suppressor on the Y chromosome and is deleted in male breast cancer

Supplementary Material

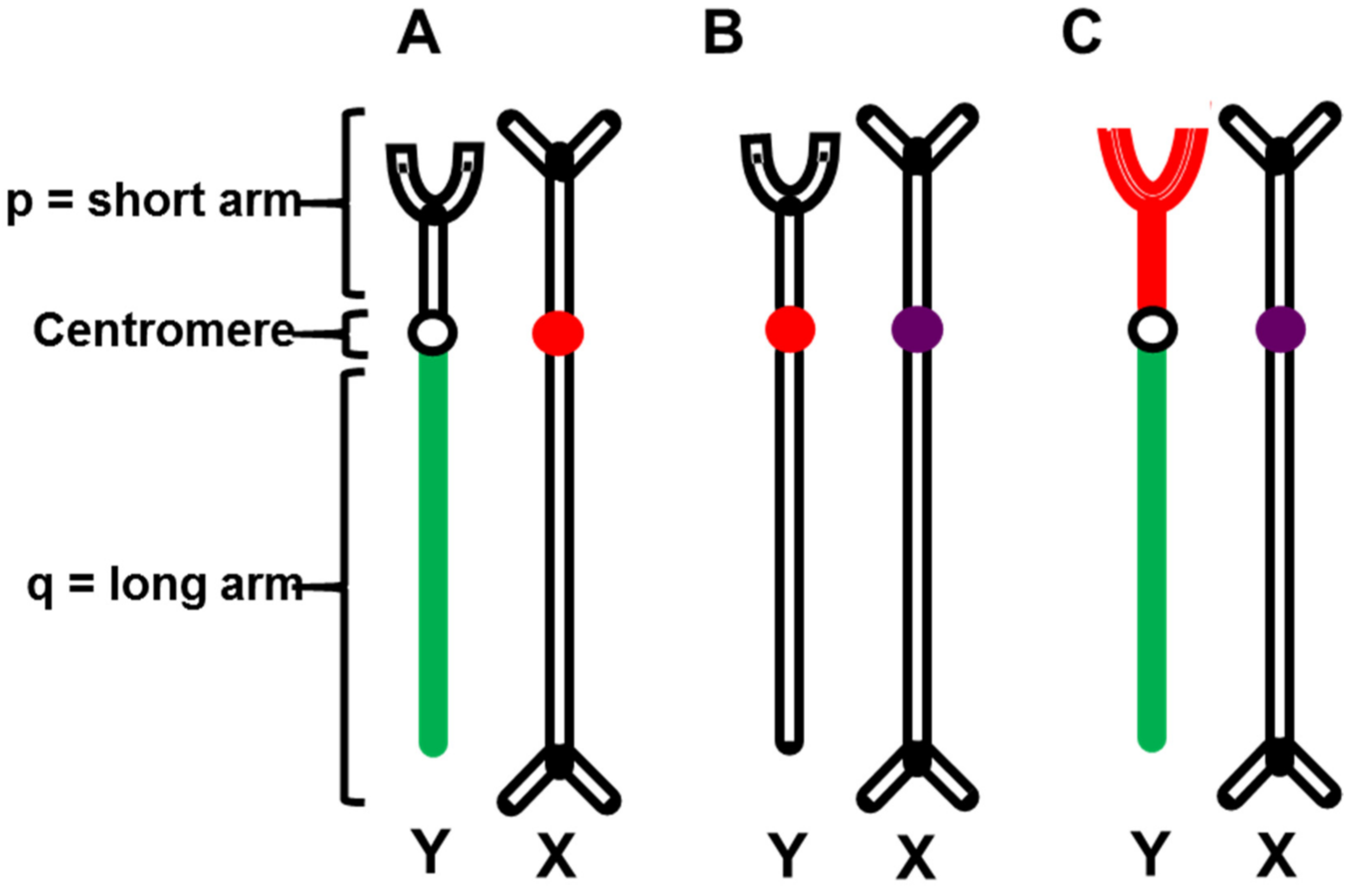

Figure S1. FISH probes used for sex chromosome enumeration. Various probes were used for enumeration of both Y and X sex chromosomes. Combinations of probes to specific regions of Y (left) and X (right) were used to assess complete Y loss using various fluorescent probe colors including **A)** green for the q-arm of the Y chromosome and red for the centromeric region of the X chromosome, **B)** red for the centromeric region of the Y chromosome, and violet for the centromeric region of the X chromosome, and **C)** green for q-arm and red for the p-arm of the Y chromosome, and violet for the centromeric region of the X chromosome.

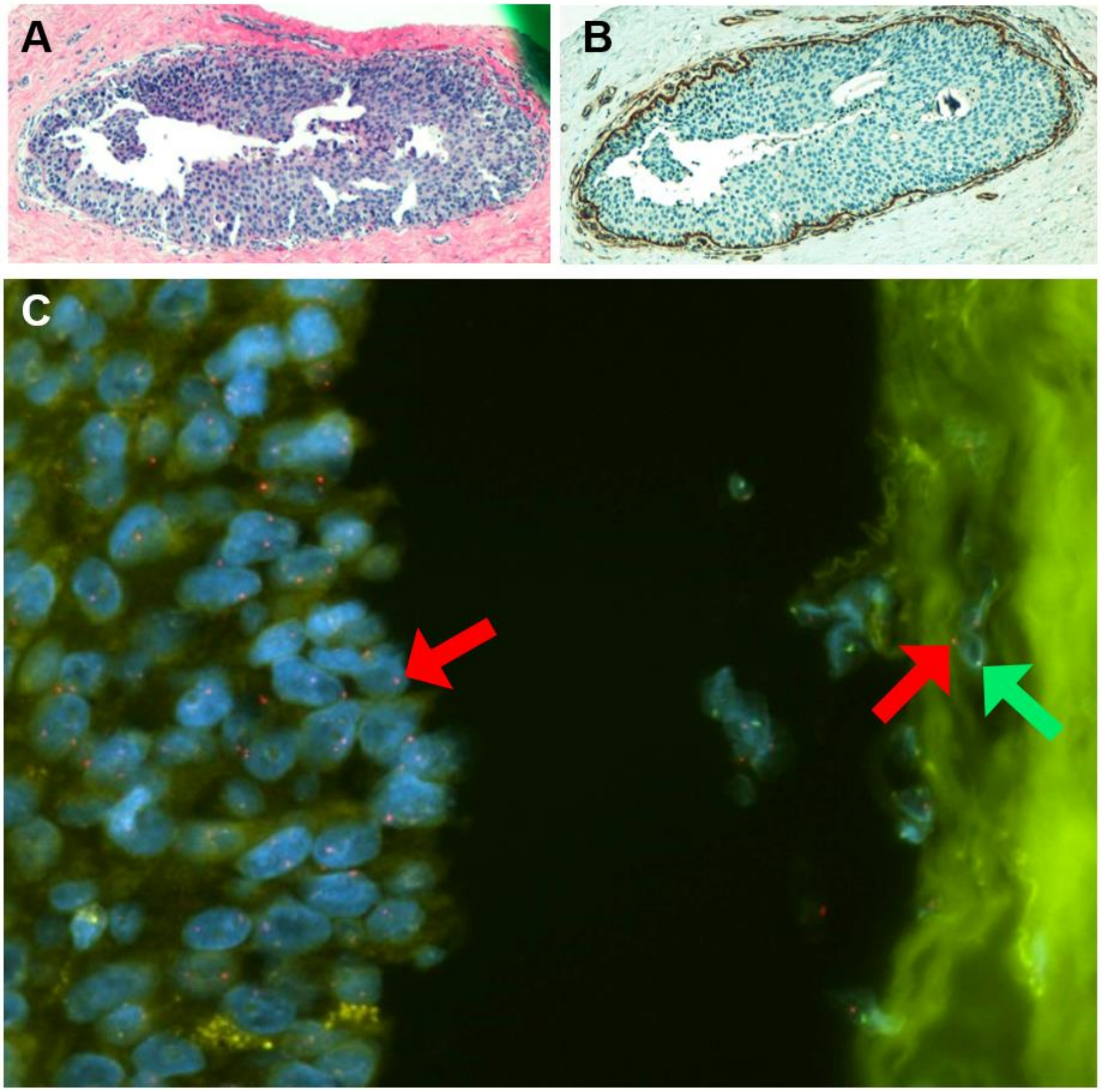

Figure S2. Clonal loss of Y chromosome in male breast DCIS. A) Hematoxylin and eosin labeling of a DCIS lesion from a male breast cancer patient with Y chromosome loss in his corresponding cancer tissue. **B)** Positive anti-smooth muscle actin labeling on a serial section of DCIS. **C)** DCIS lesion (left) with retained X chromosome FISH probe (red) and clonal loss of the Y chromosome, with an internal control in adjacent normal tissue showing the Y chromosome (right) by the presence of a green FISH probe. Red arrows point to the X probe signal and green arrow points to the Y probe signal. Nuclei are labeled with DAPI. Original magnification: 20X.

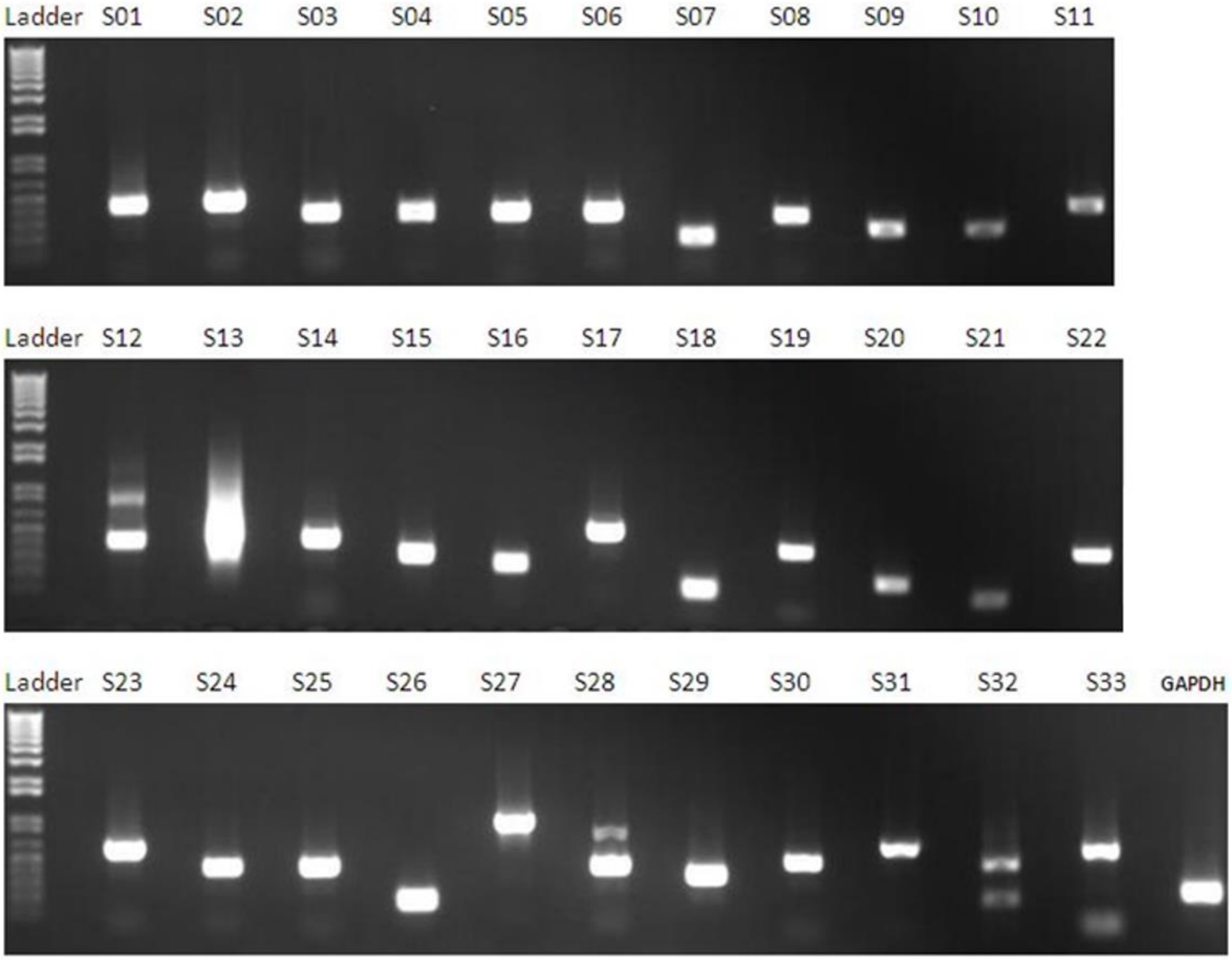

Figure S3. PCR amplicons of Sequence-Tagged Sites PCR (STS-PCR) in the Y chromosome. Thirty-three pairs of MSY Breakpoint Mapper (MSY-BP) primers, S01 to S33, were used to amplify sequence-tagged sites, and subjected to gel electrophoresis. Shown is a representative PCR performed on normal control male gDNA. A 1KB plus ladder is shown along with a GAPDH amplicon as a positive control.

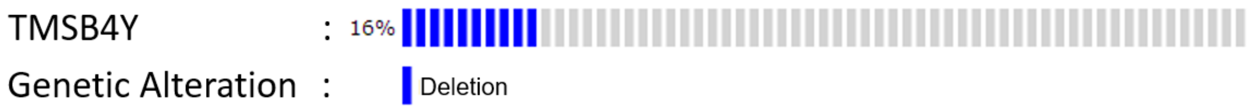

Figure S4. cBioPortal database analysis shows *TMSB4Y* deletion in metastatic prostate adenocarcinomas. A query with “TMSB4Y: HOMDEL” in the cBioPortal database yielded a data set that shows TMSB4Y deletion in 16% (10/59) of metastatic prostate tumors.

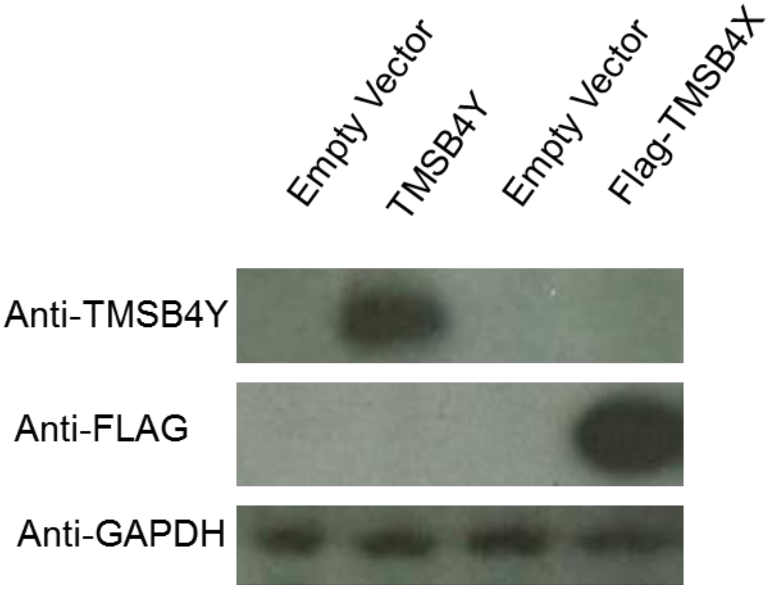

Figure S5. The anti-TMSB4Y antibody is specific. A FLAG-tagged *TMSB4X* cDNA and a *TMSB4Y* cDNA were transiently transfected separately into HEK293 cells and lysates harvested after 48 hours for western blot analysis using anti-TMSB4Y and anti-FLAG antibodies. GAPDH serves as a loading control.

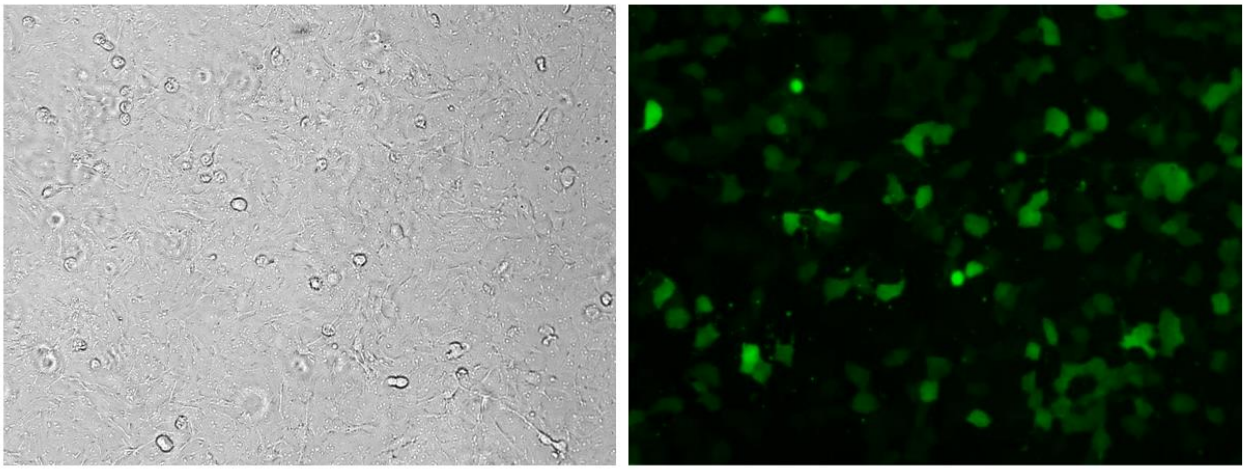

Figure S6. Transient expression of GFP in MCF-10A cells is not toxic. MCF-10A cells were transfected with a GFP expressing plasmid and observed after 48 hours. Phase contrast (left) and fluorescence (right) microscopy was used to visualize the cells. Original magnification: 20X.

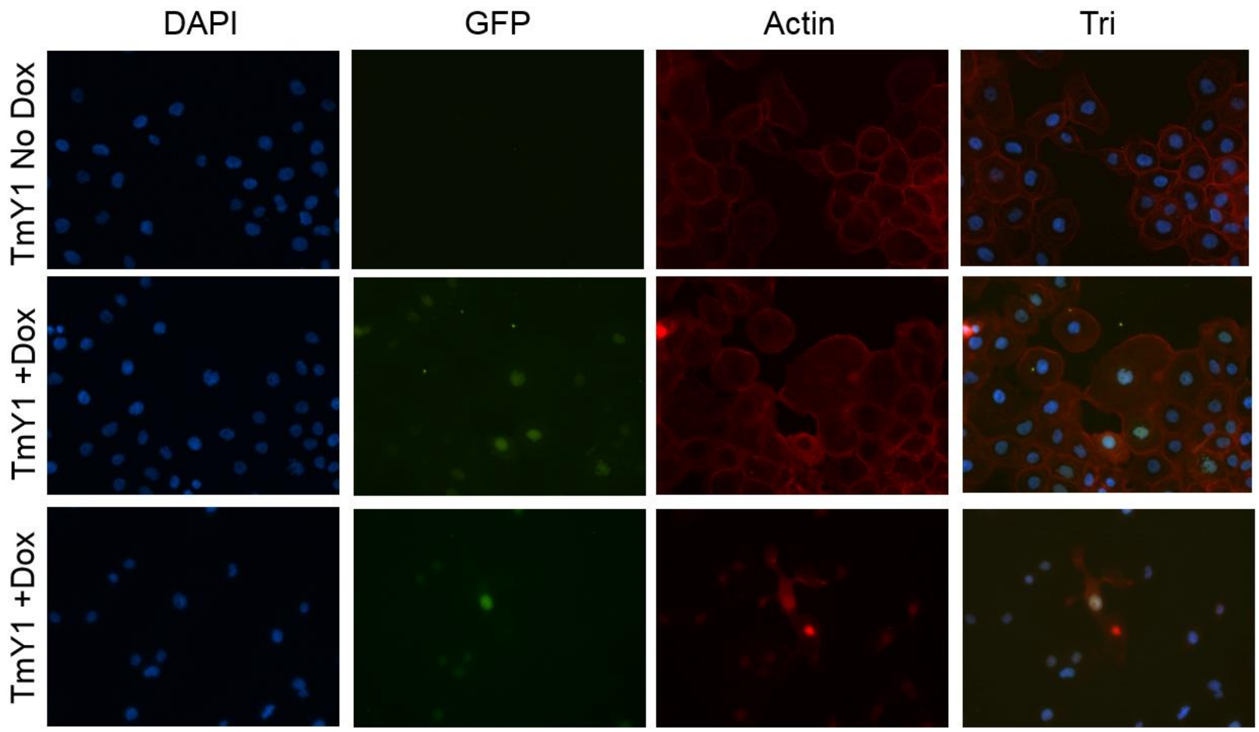

Figure S7. Dox-induction expresses TMSB4Y in Dox-inducible clones and results in aberrant morphological changes. F-actin labeling of TmY1 shows the aberrant morphological changes after Dox-induction. Blue, DAPI; Green, GFP; Red, Actin; Tri, merged. Original magnification: 20X.

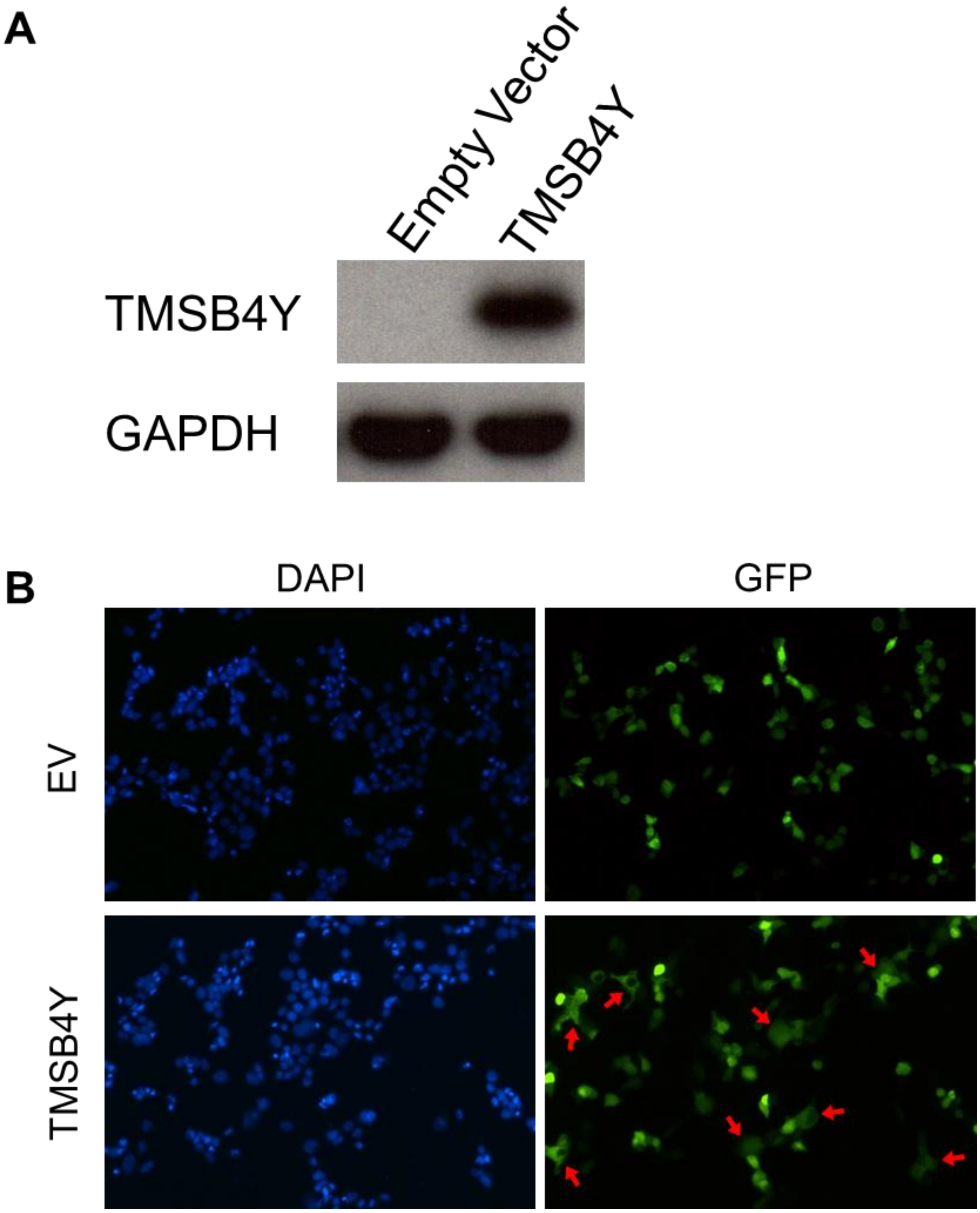

Figure S8. TMSB4Y leads to morphological changes in MCF-7 cells. MCF-7 cells were transiently co-transfected with GFP and an empty vector (EV) or a TMSB4Y-expressing vector (TMSB4Y) and assessed after 48hrs. **A)** Western blot was used to assess transient expression of TMSB4Y. GAPDH is shown as a loading control. **B)** GFP images of EV and TMSB4Y show that TMSB4Y expression led to aberrant morphological changes (red arrows) in MCF-7 cells. Blue, DAPI; Green, GFP. Original magnification: 20X.

Table S1. STS PCR primers used in this study.

S/No.	STS Identifier	Primer 1	Primer 2
S01	sY1247	GAACTCTGCAAACCTCCTGG	TTTTGAGGCGGAGTCTCG
S02	sY14	GAATATTCCCGCTCTCCGGA	GCTGGTGCTCCATTCTTGAG
S03	sY274	TTAAGGGGACAGTATTTCAACTTC	CCACATTTAAACTGAGTACAGTCC
S04	sY238	AACAAGTGAGTTCCACAGGG	GCAAAGCAGCATTCAAAACA
S05	sY1254	GACCAATTTGTCTTTGTTGCG	GCTGCTGAAGTCGGCGTA
S06	sY1240	GGGTCCTAGATAGGCTCCAAG	TTCATGTTGGCAGTGATTGG
S07	sY276	CCTACCGCATCAGTGAATTTC	TCTGTATGTGGAGTACACATGG
S08	sY1238	GGTGTGCTAACATTGCATGG	TTTGTTCCATTTCAGAGCGA
S09	sY637	CCTGCCTTTTTTAGTTTCCAGCA	TACTGTGATAGGTAGAATAATGGC
S10	sY1319	ACCTGTCTGGGAAACACCTC	GAGCCCTACAACCAGCTTCA
S11	sY1250	TTTTTCTAACCTTGCCTGCG	TGCAGAGAAGCAGCCTACAA
S12	sY1251	GACTGGAGTGGAACGGTCTC	TCACTTCCCTCCGATTTTCT
S13	sY1317	GAGATTACAGGCATGCACCA	CCACACTTAGCCCACAGTCA
S14	sY1316	AAGGCAGGTCTGATGCATGT	AAAGAAAGCTGCCTCATAGCA
S15	sY1234	TTACCCCTTTCACCCACTGA	CCATAAACTACACAAGGACGAACT
S16	sY1231	TTGCACCCGTAGTCAAATGT	ACCCACAACTCAAATCGTCT
S17	sY1230	CTCTTCCAAGCCAGCCTTTA	AACCTTTGCAAGCCACATTC
S18	sY90	CAGTGCCCCATAACACTTTC	ATGGTAATACAGCAGCTCGC
S19	sY1239	CCTAGCTCTCTTTTTCTTGCAG	CAAATATCGCCAGTGAGGCT
S20	sY210	ATCACTTGGCAGCTTTTCC	GCACTGCAACTTTTATGCCT
S21	sY121	AGTTCACAGAATGGAGCCTG	CCTGTGACTCCAGTTTGGTC
S22	sY1322	TGGAAACATTCTCAACAGGGA	GGCATTTCTCGCATGAGTTT
S23	sY280	AACTGTACTCCTGGGTAGCCTG	CTCCCGTGGGGATGAAGATAATA
S24	sY1233	TCTCCGGTATCCTGATGGAG	AAATAGGGCATTCCCAGCTC
S25	sY1682	GGTTGCACCGTAAAAGGAGA	GTCTGTCAAGACAGCGTCCA
S26	sY142	AGCTTCTATTCGAGGGCTTC	CTCTCTGCAATCCCTGACAT
S27	sY1258	AACCCCATCTCTAGCAAAAATATG	TAGGTGACAGGGCAGGATTC
S28	sY1197	TCATTTGTGTCCTTCTCTTGGA	CTAAGCCAGGAACTTGCCAC
S29	sY1191	CCAGACGTTCTACCCTTTCG	GAGCCGAGATCCAGTTACCA
S30	sY1291	TAAAAGGCAGAACTGCCAGG	GGGAGAAAAGTTCTGCAACG
S31	sY1201	CCGACTTCCACAATGGCT	GGGAGAAAAGTTCTGCAACG
S32	sY1166	AGTCGGAGTCGGAGTGTGAT	ATTCCATTGCTTTCCATTGC
S33	sY1273	GAGCTGCAACATAACAGGCA	AGGGGAACATCACACTCTGG
